# Role of Systemic Therapy in Localized Renal Cell Carcinoma: Where Do We Stand and Where Are We Heading?

**DOI:** 10.3390/cancers17101656

**Published:** 2025-05-14

**Authors:** Deepa Raghavan, Viktoriya Gibatova, Nikhil Vojjala, Nagaishwarya Moka, Aihua Edward Yen

**Affiliations:** 1 School of Medicine, Wayne State University, Detroit, MI 48201, USA; deeparaghavan@wayne.edu; 2 School of Medicine, Ross University, Miami, FL 33027, USA; viktoriyagibatova@mail.rossmed.edu; 3 Department of Internal Medicine, Trinity Health Oakland Hospital, Pontiac, MI 48341, USA; nikhil.vojjala@trinity-health.org; 4 Department of Hematology Oncology, Appalachian Regional Health, Middlesboro, KY 40965, USA; 5 Department of Hematology Oncology, Dan L Duncan Cancer Center, Baylor College of Medicine, Houston, TX 77030, USA; aihua.yen@bcm.edu

**Keywords:** localized renal cell cancer, targeted therapy, immunotherapy, perioperative therapy

## Abstract

The effectiveness of immunotherapy and targeted therapy has been well established in metastatic renal cell cancer (mRCC). These therapies demonstrated higher overall response rates and led to prolonged survival. In contrast, in localized RCC, conventional treatment is either partial or complete nephrectomy. While surgery is a curative option in early stages, high recurrence rates remain a concern, with survival rates ranging from 53% to 85%, depending on the initial stage at the time of diagnosis. Given favorable outcomes with systemic therapies in the metastatic setting, there has also been an increased interest in utilizing these therapies for the localized stage with the rationale to eradicate the micro-metastatic clone, thereby reducing the recurrence rates. Despite these encouraging developments, challenges regarding the optimal timing, duration, and combination of systemic therapies are still under investigation. Adding to that, balancing the benefits of systemic therapies with potential toxicities is also crucial, especially in patients who might otherwise benefit from surgery alone. This review describes the current landscape, ongoing clinical trials, and future directions of systemic therapy in the management of localized RCC.

## Simple Summary

Renal cell carcinoma, known as kidney cancer, is one of the common cancers that are treated with surgery alone in early stages. There has been significant progress in the field of cancer research with the development of new drugs that activate the immune system and help kill the cancer cells, called immunotherapy. It has been demonstrated that immunotherapy has been effective in late-stage kidney cancer. However, recently, clinical trials have shown its use in the early stages. Through this review article, we aim to describe the current literature and future directions of immunotherapy and targeted therapy in the treatment of early-stage (localized) renal cell carcinoma.

## 1. Introduction

Renal cell carcinoma (RCC) remains a significantly prevalent cancer and comprises 4.1% of all new cancers diagnosed in the United States [1]. In 2021, an estimated 646,960 people were living with cancers of the kidney and renal pelvis. The projected new cases in 2024 exceed 80,000 with over 14,000 deaths (17.6%)—a staggering number [1]. The 5-year relative survival rate between 2014 and 2020 was reported to be around 78%. However, the 5-year survival for localized RCC at diagnosis was notably greater at 93.3%. Additionally, even after treatment with resection, 25–30% of cases recur [2]. The incidence of RCC peaks between 60 and 70 years of age, affecting more males than females [3]. Nonetheless, RCC continues to have a significant impact on survival and overall health.

Currently, surgery with partial resection for small renal masses (≤4 cm), cT1a tumors, as well as cT1b tumors (and in selected cases–cT2a tumors), when technically feasible, and radical nephrectomy for large renal masses (>4 cm) is the first-line treatment for localized RCC, i.e., cancer that is confined to the kidney alone [4]. Other treatment options for localized RCC include ablative techniques or active surveillance [5]. While the role of systemic therapy in stage IV RCC using immunotherapy or targeted therapy is very well recognized, its application in localized disease is still evolving. This review aims to investigate and define the role of targeted therapy and immunotherapy and their use in neoadjuvant and adjuvant settings in treating localized RCC.

## 2. Methodology

We conducted a comprehensive literature search of the following electronic databases: PubMed, Medline, Embase, and Google Scholar. The search strategy included the following query: ((renal) OR (renal cell) OR (kidney)) AND ((tumor) OR (cancer) OR (carcinoma) OR (neoplasm) OR (malignancy)) AND ((local) OR (localized) OR (confined) OR (stage I) OR (stage II) OR (stage III)) AND ((adjuvant) OR (neoadjuvant) OR (targeted therapy) OR (immunotherapy)). The timeframe for inclusion was set from inception to 28 February 2025 and restricted to include the literature of stage I-III RCC. Metastatic RCC was excluded from our review.

## 3. Current Standard of Care for Management of Localized RCC

The National Comprehensive Cancer Network (NCCN) has published guidelines directing the treatment approach for kidney cancer [5]. The recommendations are stage-dependent and differ from stage I to stage IV RCC, with staging based on the TNM system [5]. In this review, we focus on and include specific standards for stages I through III only. The current recommendations for stage I RCC are partial nephrectomy, ablative techniques, active surveillance, or radical nephrectomy depending on specific patient circumstances. For stage II and III RCC, the guidelines advocate for partial or radical nephrectomy only, followed by adjuvant immunotherapy or surveillance, guided by pathological analysis. Pembrolizumab is the recommended immunotherapy, specifically for histologically clear cell renal carcinoma. Immune-oncology (IO) therapy with combination regimens can also be considered for relapsed disease.

The follow-up guidelines after primary treatment are also stage- and initial treatment-dependent. For stage I-III RCC, the follow-up includes a thorough history and physical (H&P), laboratory tests, abdominal CT or MRI, and chest imaging. The choice of the type and timing of imaging is based on the primary cancer intervention. However, it may be individualized based on the treatment schedule, side effects, associated comorbidities, and the patient’s symptoms. Additional imaging, such as a bone scan, may also be considered. The approach for long-term follow-up (>5 years) is based on patient risk factors and response to primary or adjuvant treatment, mortality assessment, and patient preference. Laboratory and clinical evaluation should be continued annually. These current recommendations for the treatment of stage I-III RCC are summarized in Table 1.

## 4. Changing Landscape in the Management of Localized RCC

In the current standard of care, the only adjuvant systemic therapy used in the treatment of stage I-III RCC is pembrolizumab, an immune checkpoint inhibitor. However, there has been an increased interest in IO in the treatment of localized RCC. Adjuvant IO with pembrolizumab in patients with localized RCC with high-risk features (clear cell histology, stage 2 tumors with high nuclear grade or sarcomatoid features, or stage 3 and stage 4 tumors irrespective of the nuclear grade) or M1 metastatic disease with fully resected metastases demonstrated progression-free survival (PFS) and overall survival (OS) benefit in the phase III Keynote 564 trial [6]. This was the first report that systemic adjuvant therapy improved overall survival in localized RCC. Therefore, based on these findings, the Food and Drug Administration (FDA) approved adjuvant pembrolizumab therapy for the treatment of high-risk localized RCC patients. Additionally, given the high incidence of recurrence in locally advanced disease, the role of neoadjuvant therapy is aimed at further improving patient outcomes and reducing recurrence. There is currently no approved neoadjuvant chemotherapy for localized RCC outside of clinical trials. The strategy remains investigational, with more research needed for definitive guideline recommendations based on efficacy and safety profiles. Several completed and ongoing trials exploring the applicability of neoadjuvant therapy, either single or in combination form, in localized RCC are discussed below. Despite first-line therapies, 25–30% of patients continue to experience recurrence of the disease. The use of adjuvant treatment through immunotherapy or targeted therapy in the management of RCC revolves around minimizing the risk of recurrence and improving disease-free survival and overall outcomes after nephrectomy by targeting residual disease or micrometastases that may not be initially detectable. Many clinical trials continue to evaluate the safety and efficacy of adjuvant agents, such as immune checkpoint inhibitors and tyrosine kinase inhibitors.

### 4.1. Studies on Adjuvant Therapy in Localized RCC

#### 4.1.1. Immunotherapy as an Adjuvant Therapy

**KEYNOTE 564**

One of the main trials that evaluated systemic adjuvant immunotherapy was the Keynote 564 phase III double-blind, placebo-controlled trial [6]. This trial included 496 participants randomized to receive pembrolizumab and 498 to receive a placebo every 3 weeks for up to 1 year. The primary endpoint demonstrated longer disease-free survival (DFS) in pembrolizumab at 24 months. The trial demonstrated a significant improvement in overall survival (OS) at 48 months in the pembrolizumab group (91.2%) versus the placebo group (86.0%) in participants with diagnosed clear cell renal carcinoma [6]. However, pembrolizumab was associated with a higher incidence of adverse events that included fatigue (19%), pruritus (18%), hypothyroidism (17%), diarrhea (14%), and rash (14%) [6].

**IMMOTION010**

Another more recent double-blind, multicenter, IMmotion010 phase III trial investigated adjuvant immunotherapy with atezolizumab in the treatment of clear cell RCC or RCC with a sarcomatoid component with high recurrence risk [7]. In the trial, a total of 778 patients were randomized between the atezolizumab and placebo groups. The primary endpoint of DFS showed 57.2 months in the experimental arm and 49.5 months in the placebo arm (hazard ratio 0.93, 95% CI 0.75–1.15, *p* = 0.50) [7]. The most common adverse events associated with atezolizumab included fatigue (30%), nausea (22%), rash (20%), diarrhea (18%), pruritus (15%), and arthralgia (12%). Overall, this study showed no evidence of improved clinically applicable outcomes in the treatment group versus placebo [7].

**CHECKMATE 914**

**Part A**

An additional notable trial is a two-part double-blind, randomized, phase III trial investigated combination adjuvant therapy or monotherapy. In part A of the trial, 405 participants were assigned to receive adjuvant nivolumab plus ipilimumab, and 411 were assigned to the placebo arm. The primary endpoint of DFS was not reached in the nivolumab plus ipilimumab group but was shown at 50.7 months in the placebo group [8]. All grade adverse events led to trial drug discontinuation in 32% of participants; four deaths were even attributed to the study drugs. However, the most experienced side effects included fatigue (36%), pruritus (28%), rash (25%), diarrhea (22%), and nausea (20%) [8].

**Part B**

Part B of the trial included 411 patients randomized to nivolumab adjuvant therapy, 208 patients to placebo, and 206 to nivolumab plus ipilimumab combination therapy. The primary endpoint of DFS in the nivolumab versus placebo group was not met [9]. The median DFS was also not reached in either arm of the trial. The DFS at 18 months was 78.4% and 75.4% in the nivolumab and placebo groups, respectively. The most common adverse events in the nivolumab group were pruritus (24%), fatigue (23.8%), diarrhea (17.4%), arthralgias (13.2%), and headaches (12%). The most common associated events in the nivolumab plus ipilimumab group included pruritus (38.7%), diarrhea (28.9%), fatigue (28.4%), hypothyroidism (22.1%), and rash (17.6%) [9].

The results from Part A and Part B of this trial do not support the use of combination or monotherapy nivolumab as adjuvant treatment of localized RCC. Other earlier studies investigating the use of adjuvant chemotherapy in localized disease found no statistically or clinically significant difference in DFS.

A recent systematic review and meta-analysis by Riveros et al. investigating the efficacy of immunotherapy as adjuvant treatment in patients with RCC demonstrated no overall benefit in DFS. The review evaluated four main trials, involving a total of 4334 patients. The primary endpoint examined DFS and the secondary outcomes were adverse or immune-mediated events. The pooled data showed no improvement in overall DFS; however, patients with positive PD-L1 expressions as well as sarcomatoid features had significant benefits [10].

#### 4.1.2. Targeted Therapy in the Adjuvant Setting

**Figure 1:**
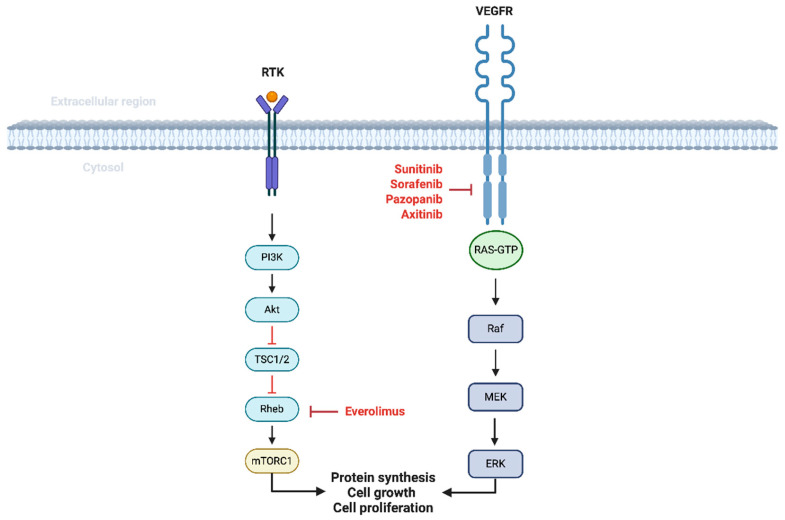
Targeted therapy in the management of RCC. VEGFR: Vascular endothelial-derived growth factor receptor pathway leads to downstream activation of RAS-GTP, increasing cell growth and proliferation. Sunitinib, Axitinib, Sorafenib, and Pazopanib are the agents that block the intracellular domain of the VEGFR. Mammalian Target of Rapamycin (MTOR) inhibitors will block the PI3K/AKT pathway, inhibiting protein synthesis and cell proliferation.

A breakthrough in the management of RCC is understanding the tumor biology itself. In the early 1990s, the discovery of recurrent loss of function of the tumor suppressor gene Von Hippel-Lindau (VHL) shifted the treatment paradigm towards targeted therapy [11]. Evidence of biallelic loss of VHL function by compound heterozygosity and promoter methylation, and the discovery of hypoxia-inducible factor-1 (HIF-1) and its link to the VHL pathway, laid the foundation to target angiogenesis in RCC [12]. Since then, many anti-angiogenic agents have been used, which later became the cornerstone in the management of metastatic RCC. In parallel, several oral multi-kinase inhibitors have also entered the clinical arena of management of RCC [13]. Mammalian target of rapamycin (mTOR) inhibitors were developed alongside vascular endothelial growth factor (VEGF) receptor-directed tyrosine kinase inhibitors for metastatic RCC. The mTOR pathway is involved in RCC promotion but also promotes angiogenesis through crosstalk with the HIF pathway. Preclinical models confirmed the antitumor activity of mTOR inhibitors, leading to the clinical development of temsirolimus and everolimus in advanced RCC [14]. Though the role of these targeted therapies is well established in the metastatic setting, there has been an increased interest in exploring these agents in patients with localized RCC. These therapies are summarized in Figure 1.

**ECOG-ACRIN E2805**

This double-blind, placebo-controlled, randomized, phase III study evaluated two VEGF multi-kinase inhibitors versus placebo. The study enrolled 1943 participants, randomized to sunitinib (647), sorafenib (649), or placebo (647), who underwent complete resection of high-risk, non-metastatic clear cell or non-clear cell RCC. The high-risk category was defined following the AJCC (American Joint Committee on Cancer)’s staging criteria as pathological stage pT1N0 high grade to pT2 (any grade) N0 and above. Patients were required to have an ECOG (Eastern Cooperative Oncology Group) performance status of 0 to 1, normal liver and hematologic function, and a creatinine clearance of at least 30 mL per minute. The primary outcome showed no significant difference in DFS among all arms of the study. Median DFS was 5.8 years for sunitinib, 6.1 years for sorafenib, and 6.6 years for the placebo group [15]. The safety profile for sunitinib included hypertension (17%), fatigue (17%), hand-foot syndrome (15%), and diarrhea (10%) as the most common incidents, while adverse events associated with sorafenib included mostly hand-foot syndrome (33%), hypertension (16%), and rash (15%). There were five deaths attributed to treatment drugs. Adjuvant treatment with sorafenib or sunitinib showed no survival benefit in the management of RCC [15].

**EVEREST**

Everolimus, an mTOR inhibitor, was investigated in another randomized, double-blind, phase III trial [16]. A total of 1545 participants with fully resected RCC with intermediate to very high recurrence risk was assigned to the everolimus (775) and placebo (770) groups. This study showed a longer recurrence-free survival (RFS) in the experimental arm with 67% compared to 63% in the placebo group at the 5-year follow-up. However, this difference was not statistically significant; therefore, there was no benefit in the adjuvant everolimus treatment over placebo. The most common side effects included mucositis (14%), hypertriglyceridemia (11%), and hyperglycemia (5%) [16].

**SORCE**

This international, randomized, double-blind, three-arm trial assessed the use of sorafenib as an adjuvant treatment of RCC at intermediate to high risk of recurrence [17]. A total of 1711 patients were randomly assigned to either 3 years of placebo, 1 year of sorafenib followed by 2 years of placebo, or 3 years of sorafenib. The primary outcome of DFS was not reached for 3 years in the sorafenib or placebo groups [17]. The restricted mean survival time (RMST) was found to be 6.81 years for 3 years of sorafenib and 6.82 years for placebo. There was no difference in overall DFS or OS among all three arms. The most common associated adverse events included hand-foot skin reactions (76%), diarrhea (45%), rash (40%), fatigue (37%), and hypertension (36%); more than half the study participants discontinued treatment [17].

Currently, the evidence for adjuvant immunotherapy or targeted therapy in localized RCC with a high risk of recurrence is discouraging. Additionally, a systematic review published by Sun et al. evaluated the use of VEGF-targeted therapy in the treatment of RCC. The review included three randomized controlled phase III trials and over 3600 participants [18]. The combined analysis of sunitinib, sorafenib, and pazopanib demonstrated that VEGF receptor-targeted therapy was not associated with improved DFS. Additionally, the adjuvant treatment group experienced significantly more grade 3–4 adverse events [18].

### 4.2. Perioperative and Neoadjuvant Therapy in Localized RCC

#### 4.2.1. Immunotherapy in the Neoadjuvant Setting

The literature on the use of perioperative (neo plus adjuvant) and neoadjuvant treatment alone in localized RCC is limited. Neoadjuvant therapy was initially explored to decrease tumor size and metastatic burden of advanced disease in candidates for surgical debulking of stage IV RCC [19]. However, there has been increasing interest in exploring this therapeutic option with the potential of lowering disease recurrence following surgical resection and improving overall oncologic outcomes.

**PROSPER EA8143**

The use of nivolumab before nephrectomy and as adjuvant chemotherapy in patients with high-risk RCC was investigated in an open-label phase III trial, PROSPER EA8143 [20]. A total of 819 patients with T2 or higher stage before resection or recently resected limited metastatic RCC were assigned to either perioperative nivolumab (404) or surgery alone (415). The primary endpoint of RFS was not statistically different between the two arms, with 33% RFS in the nivolumab group and 33% in the surgery-only group. The use of perioperative nivolumab therapy with subsequent adjuvant nivolumab therapy did not improve RFS over conventional surgical resection with surveillance only. The most common adverse effects experienced during the trial included stomatitis (61%), fatigue (45%), diarrhea (38%), rash (36%), and infections (29%) [20].

#### 4.2.2. Targeted Therapy with or Without Immunotherapy in the Neoadjuvant Setting

Although several trials have explored the use of neoadjuvant therapy in localized RCC, the PROSPER trial remains the largest, to date. Additionally, much of the prior research focused on the effects of neoadjuvant chemotherapy on tumor size reduction. Only a few of the studies assessed its effects on survival and disease recurrence.

**NEOAVAX**

This phase II, single-arm trial evaluated axitinib plus avelumab as neoadjuvant therapy in patients with localized RCC who are at high risk of relapses after surgery [21]. The trial recruited 40 patients with the primary endpoint of the partial response rate of the primary tumor. The partial response was achieved in 30% of participants, with 32% experiencing disease recurrence at the 8-month follow-up. The secondary outcomes of DFS and OS were not reached [21]. The most common adverse events associated with treatment included diarrhea (40%), fatigue (35%), hypertension (30%), hand-foot skin reactions (25%), and nausea (20%) [21].

**PADRES**

The response of the tumor to axitinib before partial nephrectomy was assessed in PADRES, a single-arm phase II clinical trial [22]. The 27 enrolled patients had localized RCC and required partial nephrectomy for nephron preservation and received 8 weeks of axitinib before surgery. The primary outcome was successful partial nephrectomy completion. Axitinib reduced tumor size and complexity and enabled partial nephrectomy in 20 patients and negative margins in 25 patients. The side effect profile mainly included diarrhea (37%), fatigue (33%), hypertension (30%), hand-foot skin reactions (26%), and nausea (22%). Neoadjuvant axitinib resulted in reductions in tumor size and complexity, making partial nephrectomies and preservation of renal function possible [22].

Additionally, a phase II trial by Huang et al. explored the combination of immunotherapy and targeted therapy, toripalimab and axitinib, in locally advanced RCC in the neoadjuvant setting [23]. The objective response rate was shown to be 45%, while the secondary outcome of median DFS was not reached. However, the estimated DFS rates for 1 year and 2 years were 84.7% and 84.7%, respectively. Diarrhea (37%) was the most common adverse effect, followed by fatigue (33%), hypertension (30%), and hand-foot skin reactions (26%) [23].

A real-world retrospective review of neoadjuvant targeted therapy was assessed in a study by Silberstein et al. This retrospective review included 12 patients who required nephron-sparing surgery and received sunitinib before surgery. The data demonstrated a decrease in tumor size in all of the patients, with a mean reduction in maximum diameter of 1.5 cm (21.1%) [24] (Table 2).

### 4.3. Future Directions

Even with the development and recent use of adjuvant chemotherapy for RCC, many patients continue to experience disease recurrence after surgical intervention. Much of the currently available published literature presents conflicting results. More studies are needed to assess the value of immunotherapy and targeted therapy in decreasing disease recurrence and survival. Additionally, the data on neoadjuvant treatment are available on a small scale, thus, larger trials are needed to establish its utility. The efficacy and use of systemic treatment in the setting of localized, stage I-III RCC are under active investigation. Several new ongoing studies with active patient enrollment were additionally summarized in Table 3, which aims to further evaluate systemic therapy, targeted therapy, and immunotherapy in terms of overall mortality reduction and survival benefits. However, future studies should continue to focus on the development of risk stratification strategies, perhaps guided by specific molecular signatures as well as a systematic approach to patient selection for neoadjuvant and adjuvant therapy options.

## 5. Conclusions

In summary, the usage of neoadjuvant and adjuvant therapies in patients with high-risk localized RCC is the future, focusing on achieving a high tumor response, minimizing recurrence rates, and improving long-term outcomes. Clinicians are encouraged to communicate with their patients regarding the available data and highlight unresolved questions to facilitate informed decision-making about the personalized application of systemic therapy in patients with localized RCC. There is room for further research particularly biomarker-driven personalized treatment strategies by integrating PD-L1 expression, genomic features, and clinicopathological parameters to establish a multidimensional risk stratification model. This would enable precise patient selection in adjuvant/neoadjuvant settings as well. Subsequent clinical trials should prioritize randomized double-blind designs with expanded cohort diversity (e.g., non-clear cell subtypes and low-risk populations), while systematically evaluating the long-term efficacy and safety of combination regimens (e.g., immune checkpoint inhibitors with TKIs or dual immunotherapy approaches). Additionally, unresolved critical clinical issues—including optimal treatment duration, resistance mechanisms, and cost-effectiveness analyses—require in-depth exploration to inform evidence-based guideline updates.

## Figures and Tables

**Table 1:** RCC TNM staging and NCCN recommendations for the management of stage I-III RCC.

RCC Stage	Description	TNM	Primary Management	Secondary Management	Follow-Up
Stage I	Tumor < 7 cm and has not spread outside of the kidney	T1, N0, M0	Partial nephrectomy (preferred), ablative techniques, radical nephrectomy, or active surveillance	Surveillance	Active surveillance: - H&P (annually) - Laboratory testing (annually) - Abdominal CT/MRI (within 6 months), followed by CT/MRI/US (annually) - Chest imaging (chest X-ray/CT at baseline, then annually) Ablative techniques: - H&P (annually) - Laboratory testing (annually) - Abdominal CT/MRI/US (at 1–3 mo, 6 mo, 12 mo, then annually) - Chest imaging (X-ray/CT annually for 5 years) Partial/radical nephrectomy: - H&P (annually) - Laboratory testing (annually) - Abdominal CT/MRI (within 3–6 mo, then annually for 5 years) - Chest imaging (chest X-ray/CT annually for 5 years)
Stage II	Tumor > 7 cm and has not spread outside of the kidney	T2, N0, M0	Partial nephrectomy or radical nephrectomy	Non-clear cell: surveillance. Clear cell: surveillance or adjuvant pembrolizumab (category 1)	- H&P (annually) - Laboratory testing (annually) - Abdominal CT/MRI (every 6 mo for 2 years, then annually for 5 years) - Chest X-ray/CT (for at least 5 years)
Stage III	Cancer has spread to adjacent tissue, may involve lymph nodes, and no distant metastasis	T3, N0, M0 or T1-T3, N1, M0	Radical nephrectomy or partial nephrectomy (if indicated)	Non-clear cell: surveillance or clinical trial. Clear cell: adjuvant pembrolizumab (category 1) or surveillance	- H&P (every 3–6 months for 3 years, then annually for 5 years) - Comprehensive metabolic panel (every 3–6 months for 3 years, then annually for 5 years) - Abdominal CT/MRI (baseline within 3–6 mo, then CT/MRI/US every 3 mo for 3 years, then annually for 5 years) - Chest imaging (baseline CT within 3–6 mo, then every 3–6 mo for 3 years and annually for 5 years)

TNM: Tumor, Node and Metastasis Staging; NCCN: National Comprehensive Cancer Network; RCC: Renal Cell Cancer; H&P: History and Physical; CT: Computerized tomography; MRI: Magnetic Resonance Imaging; US: Ultrasound.

**Table 2:** Summary of adjuvant and neoadjuvant therapies in localized RCC.

Trial Name	Year	Design	Number of Participants	Intervention	Type	Results
ECOG-ACRIN E2805 [15]	2016	Randomized, phase III	1943	Sunitinib, sorafenib	Adjuvant	Median DFS was 5.8 years for sunitinib, 6.1 years for sorafenib, and 6.6 years for placebo
S-TRAC [25]	2016	Randomized, phase III	615	Sunitinib	Adjuvant	Median DFS was 6.8 years for sunitinib and 5.6 years for placebo
PROTECT [26]	2017	Randomized, phase III	1538	Pazopanib	Adjuvant	Median DFS was 54 months for the placebo and not attained for the pazopanib group
ATLAS [27]	2018	Randomized, phase III	724	Axitinib	Adjuvant	No difference in DFS [hazard ratio (HR) = 0.870; 95% confidence interval (CI): 0.660–1.147, *p* = 0.321]
ARISER [28]	2017	Randomized, phase III	864	Girentuximab	Adjuvant	Median DFS was 71.4 months forgirentuximab and not reached for placebo
SORCE [17]	2020	Randomized, phase III	1711	Sorafenib	Adjuvant	Ten-year DFS rate was 53% for 3-year sorafenib, 55% for 1-year sorafenib and 54% for placebo
EVEREST [16]	2022	Randomized, phase III	1499	Everolimus	Adjuvant	6-year RFS estimate was 64% for Everolimus and 61% for placebo
IMmotion010 [7]	2022	Randomized, phase III	778	Atezolizumab	Adjuvant	Median DFSl was 57.2 months for atezolizumab and 49.5 months for placebo
CheckMate 914 (Part A) [8]	2023	Randomized, phase III	816	Nivolumab, ipilimumab	Adjuvant	Median DFS was not reached for nivolumab plus ipilimumab and was 50.7 months for placebo
CheckMate 914 (Part B) [9]	2025	Randomized, phase III	825	Nivolumab	Adjuvant	Median DFS was not reached in either arm, DFS probabilities were 83.3% in nivolumab and 78.2% in placebo (at 12 months)
Keynote 564 [6]	2022	Randomized, phase III	994	Pembrolizumab	Adjuvant	DFS at 24 months was 77.3% for Pembrolizumab and 68.1% for placebo
Silberstein et al. [24]	2010	Retrospective	12	Sunitinib	Neoadjuvant	All patients had a decrease in size with a mean reduction in maximum diameter of 1.5 cm (21.1%)
Karam et al. [29]	2014	Phase II	24	Axitinib	Neoadjuvant	The median reduction in renal tumor diameter was 28.3%
Rini et al. [30]	2015	Phase II	25	Pazopanib	Neoadjuvant	R.E.N.A.L. score decreased in 71% of tumors and 92% had a reduction in tumor volume
Zhang et al. [31]	2015	Retrospective	18	Sorafenib	Neoadjuvant	Tumor size decreased from 7.8 cm to 6.2 cm and the median value of average tumor CT value decreased from 61 HU to 52 HU
Hatiboglu et al. [32]	2017	Prospective	12	Sorafenib	Neoadjuvant	Primary renal tumor diameter changed from 5.4 cm to 4.4 cm for sorafenib group and 10.6 cm to 10.7 cm in placebo group
NEOAVAX [21]	2019	Phase II	40	Axitinib, avelumab	Neoadjuvant	Median tumor size reduction was 20% with 32% experiencing recurrence, median OS was not reached
Lebacle et al. [33]	2019	Phase II	18	Axitinib	Neoadjuvant	Primary tumor diameter had a median size reduction of 17%
PADRES [22]	2023	Phase II	26	Axitinib	Neoadjuvant	Decreased tumor size (7.7 to 6.3 cm) and RENAL score (11 vs. 10, *p* < 0.001)
Carlo et al. [34]	2023	Phase II	18	Nivolumab	Neoadjuvant	Median RFS at 1 year was 82% (95% CI 65–100%)
Huang et al. [23]	2024	Phase II	18	Toripalimab, axitinib	Neoadjuvant	The objective response rate was 45%, median DFS was not reached, and estimated DFS rates at 1 year and 2 years were 84.7% and 84.7%
PROSPER EA8143 [20]	2024	Randomized, phase III	819	Nivolumab	Neoadjuvant	33% had RFS in nivolumab versus 33% in surgery only

**Table 3:** Ongoing trials investigating adjuvants or neoadjuvant therapy in localized RCC.

Trial Name	Year	Design	Number of Participants	Intervention	Type	Results
SPARC-1 (NCT04028245)	2019	Open-label pilot	Recruiting	Spartalizumab, canakinumab	Neoadjuvant	Pending
RAMPART (NCT03288532)	2021	Multi-arm multi-stage, phase III	Recruiting	Durvalumab, tremelimumab	Neoadjuvant	Pending
LITESPARK-022 (NCT05239728)	2022	Randomized, phase III	Recruiting	Pembrolizumab, belzutifan	Adjuvant	Pending
NESCIO (NCT05148546)	2022	Randomized, phase II	69	Nivolumab, ipilimumab, relatlimab	Neoadjuvant	Pending
TUOAD-RCC (NCT06584435)	2022	Phase II	Recruiting	Teprolizumab	Adjuvant	Pending
Narayan et al. (NCT05733715)	2023	Randomized pilot	Recruiting	Pembrolizumab, lenvatinib	Neoadjuvant	Pending
INTerpath-004 (NCT06307431)	2024	Randomized, phase II	Recruiting	V940, pembrolizumab	Adjuvant	Pending
MRD GATE RC(NCT03142334)	2024	Multicenter open label	Recruiting	Pembrolizumab	Adjuvant	Pending
Voss et al. (NCT03005782)	2025	Phase II	Recruiting	Cemiplimab, fianlimab	Neoadjuvant	Pending
Liu et al. (NCT06574412)	2025	Phase II	Pending	Cardonilizumab, renvastinib	Adjuvant and neoadjuvant	Pending
